# Anatomical correlation between left atrium pulmonary vein ablation targets of atrial fibrillation and adjacent bronchi and pulmonary arteries by MSCT

**DOI:** 10.1186/s12872-021-01881-2

**Published:** 2021-02-10

**Authors:** Yan-Jing Wang, Huan Sun, Xiao-Fei Fan, Meng-Chao Zhang, Ping Yang, Hong Zeng, Lin Liu

**Affiliations:** 1 Radiology Department, China-Japan Union Hospital of Jilin University, 126 Xiantai Street, Changchun, 130031 Jilin Province China; 2 Cardiology Department, Cardiovascular Institute of Jilin Province, China-Japan Union Hospital of Jilin University, 126 Xiantai Street, Changchun, 130031 Jilin Province China; 3 Jilin Provincial Precision Medicine Key Laboratory for Cardiovascular Genetic Diagnosis, Changchun, 130031 Jilin Province China

**Keywords:** Computed tomography, Atrial fibrillation, Left atrial roof, Bronchi, Pulmonary artery

## Abstract

**Background:**

The ablation targets of atrial fibrillation (AF) are adjacent to bronchi and pulmonary arteries (PAs). We used computed tomography (CT) to evaluate the anatomical correlation between left atrium (LA)-pulmonary vein (PV) and adjacent structures.

**Methods:**

Data were collected from 126 consecutive patients using coronary artery CT angiography. The LA roof was divided into three layers and nine points. The minimal spatial distances from the nine points and four PV orifices to the adjacent bronchi and PAs were measured. The distances from the PV orifices to the nearest contact points of the PVs, bronchi, and PAs were measured.

**Results:**

The anterior points of the LA roof were farther to the bronchi than the middle or posterior points. The distances from the nine points to the PAs were shorter than those to the bronchi (5.19 ± 3.33 mm vs 8.62 ± 3.07 mm; *P* < .001). The bilateral superior PV orifices, especially the right superior PV orifices were closer to the PAs than the inferior PV orifices (left superior PV: 7.59 ± 4.14 mm; right superior PV: 4.43 ± 2.51 mm; left inferior PV: 24.74 ± 5.26 mm; right inferior PV: 22.33 ± 4.75 mm) (*P* < .001).

**Conclusions:**

The right superior PV orifices were closer to the bronchi and PAs than other PV orifices. The ablation at the mid-posterior LA roof had a higher possibility to damage bronchi. CT is a feasible method to assess the anatomical adjacency in vivo, which might provide guidance for AF ablation.

## Background

Atrial fibrillation (AF) is the most common arrhythmia in clinics worldwide. Radiofrequency or balloon cryoablation of circumferential pulmonary vein (PV) vestibule is the standard method for the treatment of AF [1]. However, in recent years, radical isolation strategies, such as left atrial roof and isthmus ablation, as well as the fragmentation potential ablation have been widely applied, the damage to the surrounding tissues caused by ablation energy is under intensive focus [2]. Hitherto, the cardiac electrophysiologists have paid enough attention to the injury of esophagus and phrenic nerves, and hence, several studies have explored the anatomical correlation between the left atrium (LA)-PV ablation targets of AF and adjacent esophagus and phrenic nerve [3–7]. In our previous studies by MSCT, we have showed a close relation of the right phrenic nerves to superior caval vein, right atrium, and right superior PV, and the adjacency of the left phrenic nerves to left atrial appendage and coronary venous tributaries [7]. Recently Felix Bourier et al. [8] reported a feasible method combining the 3D-mapping system integration module and CT-derived anatomy, showing that even the three‐dimensional image integration also can show the right phrenic nerves into fluoroscopic imaging clearly. Anatomically, the ablation targets of AF are adjacent to the lung, airway, and pulmonary artery (PA), such that the ablation energy may damage the bronchi, PAs, and lung parenchyma, resulting in the symptoms of cough, dyspnea, hemoptysis, and even death in the patients [5, 9]. Animal studies have shown that ablation energy may cause acute bronchial inflammation and mucosal injury [10]. Also, rare clinical cases of atrial-bronchial fistula caused by the ablation of AF are reported. [9] The clinical trial of sustained treatment of paroxysmal atrial fibrillation (STOP-AF) [11] showed that the incidence of persistent cough caused by cryoablation was 17%, and its subsequent post-approval clinical trial [12] showed that the incidence of hemoptysis caused by ablation was 1%. Verma et al. reported a patient with a formation of ice crystals in the left main bronchus after 50 s of ablation of the left superior PV [13]. Furthermore, the PV ablation resulted in ice crystal formation and internal hemorrhage in the main bronchus was reported in seven patients [14].

The high tissue resolution of multi-slice spiral computed tomography (MSCT) angiography can visually and accurately display the anatomy of LA and its surrounding tissues. The powerful volume rendering (VR) and multi-planar reformation (MPR) technologies of MSCT can not only display the spatial anatomical correlation between the heart and adjacent structures, but also achieve accurate quantitative measurements. Moreover, MSCT imaging is simple, quick, non-invasive, and importantly, can reconstruct and accurately measure the images at any angles in vivo, thereby avoiding tissue shrinkage, distortion, and vascular volume changes caused by the immersion of the formalin solution in autopsy. It is no doubt that MSCT is more accurate to measure the spatial anatomical distances between two adjacent structures of the heart as compared to the autopsies. Recently, we have successfully achieved the non-invasive phrenic nerve imaging in patients using MSCT angiography [7], but few studies had described the anatomical adjacency between the ablation targets of AF and the bronchi as well as the PAs. In this study, MSCT angiography was used to evaluate the spatial anatomical relation between ablation targets of AF, such as four PVs and ablation lines on the LA roof, and the adjacent bronchi and PAs in vivo, which might provide a promising guidance for cardiac electrophysiologists to reduce the complications of AF ablation.

## Methods

### Patients

A total of 126 patients were enrolled consecutively for coronary MSCT angiography examination from January to October 2017. The cohort comprised of 67 males and 59 females, aged 30–78 years (average 54.28 ± 9.28 years). Exclusion criteria: patients with allergic history of iodine contrast medium, renal insufficiency or renal failure, pregnancy, and PV malformation. This study was approved by the Ethics Committee of the China-Japan Union Hospital of Jilin University. All patients signed the written consent form before the coronary angiography examination.

### Scanning technique

All studies were performed on a Toshiba 320-row MSCT scanner (Aquilion one TSX-301A, Tokyo, Japan) with respiratory and electrocardiogram gating. Imaging parameters were as follows: tube voltage 120 kV, tube current 350–450 mA, slice thickness 0.5 mm, interlayer spacing 0.5 mm, and spin time 350 ms. The patient was connected to a binocular high-pressure injector (SCT 211, Medrad Inc. USA). The scan was set from the upper margin of the aortic arch to the diaphragmatic surface. A 20 mL of the contrast agent was injected at a rate of 3–5 mL/s (Omnipaque 350 mg/mL, GE Healthcare Inc., Marlborough, MA, USA), followed by 15 mL of saline at the same rate. An intelligent bolus tracking technique was used to trigger the scanning when the CT value of the descending aorta reached 200–220 HU. The patients hold their breath for 4–7 s, and further 40–60 mL contrast agent was infused intravenously at a flow rate of 3–5 mL/s, followed by 15 mL of saline at the same rate.

### Image post-processing and data measurement

**Fig. 1:**
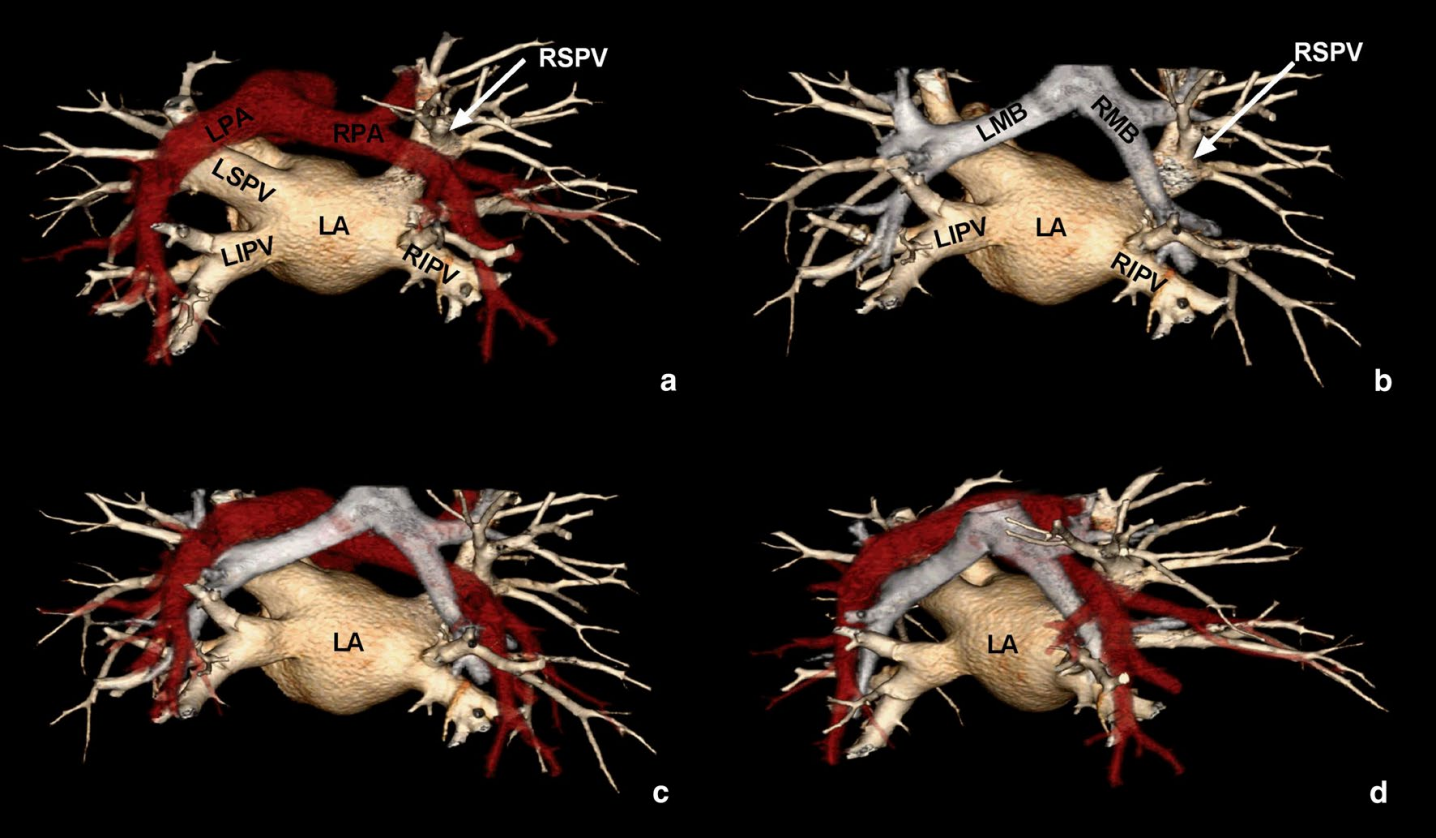
Three dimensional (3D) CT images of the left atrium (LA), pulmonary vein (PV), bronchial tree, pulmonary artery (PA), and branches. **a** 3D image of the LA, PVs and PAs. **b** 3D image of the LA, PVs, and bronchi. **c**, **d** 3D images of the LA, PVs, PAs and bronchi at different angles of view. LPA, left PA; RPA, right PA; RSPV, right superior PV; RIPV, right inferior PV; LSPV, left superior PV; LIPV, left inferior PV; RMB, right main bronchus; LMB, left main bronchus

Original images were transferred to the post-processing workstation (Vitrea; HP XW 8600 workstation, Minnetonka, MN, USA). The three-dimensional (3D) reconstruction images of the LA, four PVs, bronchial tree, PA and its branches were obtained by VR technique (Fig. 1). The MPR technique was then used to obtain multi-directional images of the LA, PVs, bronchial tree, as well as the PA and its branches. The sizes of the LA and four PV orifices, as well as, the shortest spatial distances between the adjacent structures of the heart were measured. All data were independently obtained by two radiologists with > 5 years of experience in cardiovascular imaging, and we use the averaged of the two measurements to avoid the observation errors. If the difference between the two measurements was large, re-measurement by another radiologist with > 10 years of experience in cardiovascular imaging was involved.

#### Measurement of LA and PVs

**Fig. 2:**
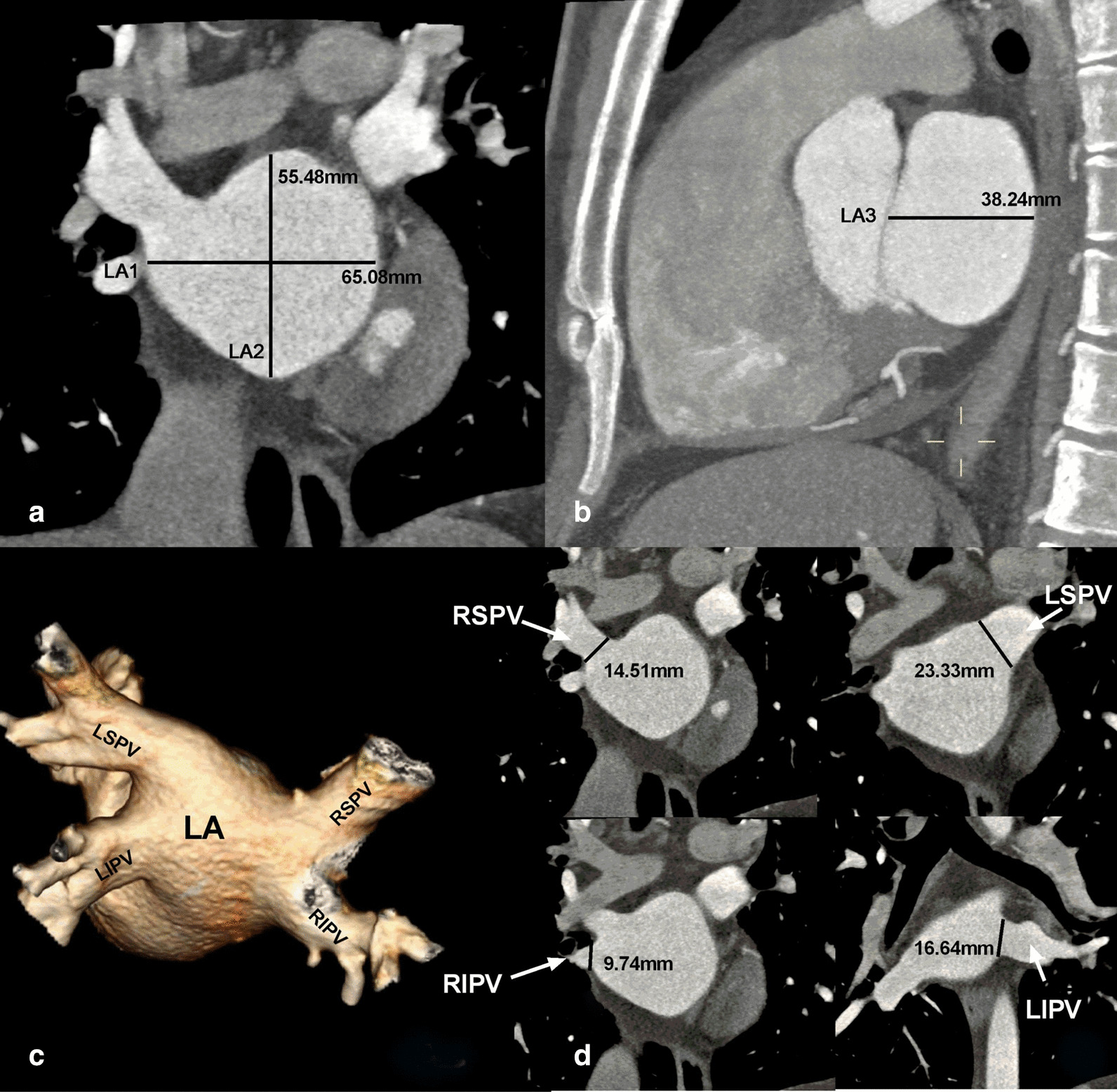
Measurement of the left atrium (LA) size and diameters of the pulmonary vein (PV) with multi-slice spiral CT. **a**, **b** Maximum diameters of the LA in coronal and sagittal views, respectively. LA1: transverse diameter; LA2: vertical diameter; LA3: anterior and posterior diameter. **c** Three dimensional images of the LA and four PVs. **d** Maximum upper and lower diameters of the four PVs measured at the coronary view. RSPV, right superior PV; RIPV, right inferior PV; LSPV, left superior PV; LIPV, left inferior PV

Firstly, we identified the LA on the MPR images, and then, the slices displaying the maximum area of the LA on the coronal and sagittal images were selected to measure the transverse diameters (LA1), vertical diameters (LA2), and anterior and posterior diameters of the LA (LA3) (Fig. 2a, b). Due to the different morphologies and large variations of the PV orifices in the axial review, we measured the maximum upper and lower diameters of the PV orifices in the coronary position (Fig. 2c, d).

#### Measurement of the shortest spatial distance between LA roof and PAs, bronchi

**Fig. 3:**
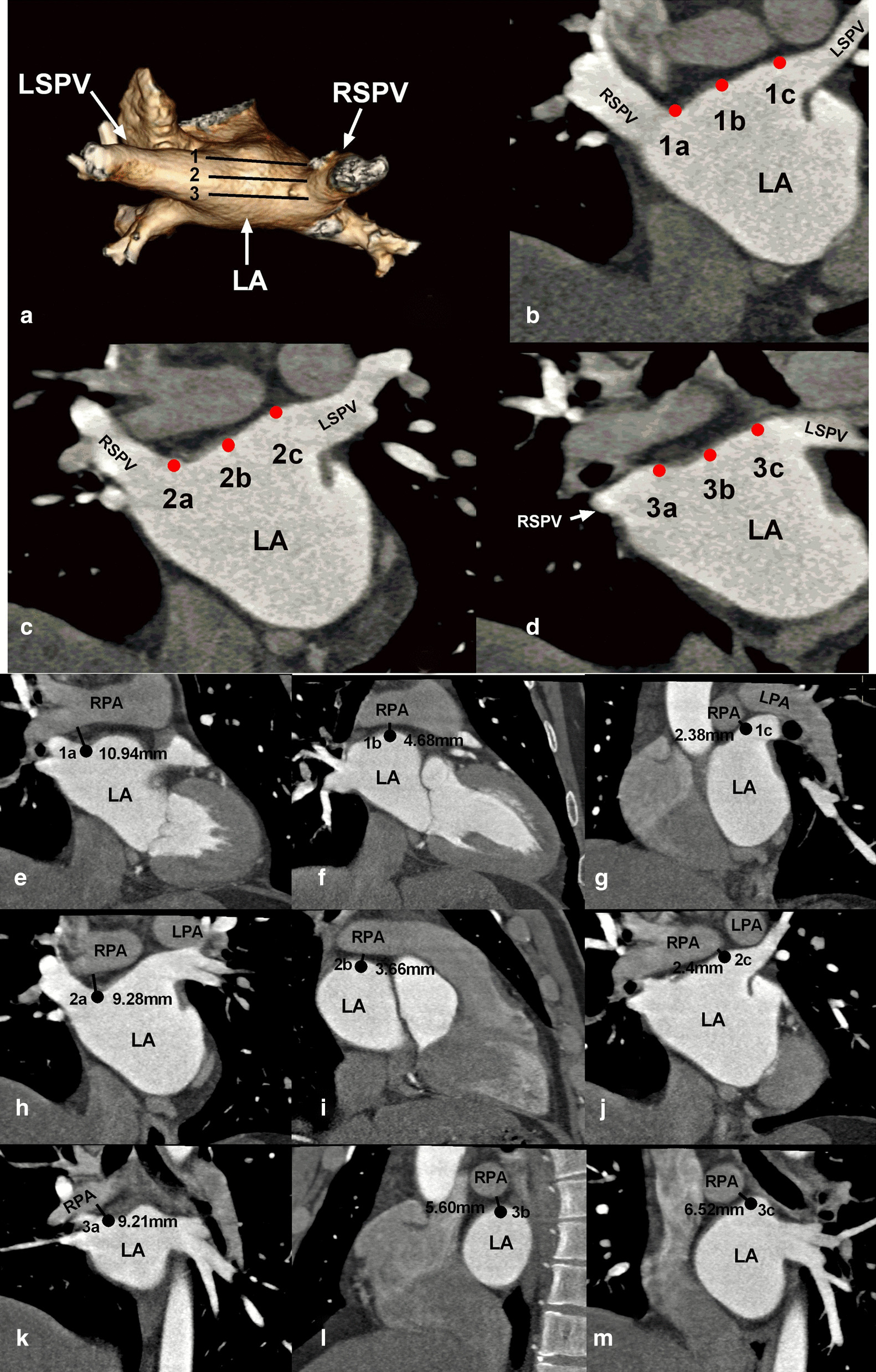
The nine points on the left atrial (LA) roof and the measurements of the shortest spatial distance between the nine points and the pulmonary arteries (PAs). **a** Three dimensional images of the LA roof and four pulmonary veins (PVs). The LA roof was divided into three layers from front to back. **b**–**d** Coronal images of the 1st, 2nd, and 3rd layers, respectively. Each layer is divided into three points (a, b, and c) from right to left. **e**–**m** Shortest spatial distances between the points of “1a” to “3c” and the adjacent PAs, respectively. LPA, left PA; RPA, right PA; RSPV, right superior PV; RIPV, right inferior PV; LSPV, left superior PV; LIPV, left inferior PV

The LA roof is defined as the uppermost region of the LA between the left and the right superior PVs. Since the structure of the LA roof is not a straight line, but a curved dome surface, we firstly outlined the scope of the LA roof on the MPR coronal views in reference to the axial and sagittal images, and then artificially divided the LA roof into three layers from front to back. The first and the third layer was separated by the line between the front and posterior edges of the left and right superior PV orifices, respectively, and the second layer was in the middle of the two layers. Each layer was further divided into three points (a, b, and c) from right to left. Point “a” was at the junction of the right superior PV and the LA roof, point “c” was at the junction of the left superior PV and the LA roof, and point “b” was in the middle of point “a” and “c”. Thus, each patient’s domed LA roof was artificially divided into 3 layers and nine points (Fig. 3a–d). In reference to the images of axial, coronal and sagittal views, the shortest spatial distances from the nine points to the adjacent PAs and bronchi and branches were measured (Fig. 3e–m).

#### Measurement of the shortest spatial distance between PV orifices and PAs, bronchi

**Fig. 4:**
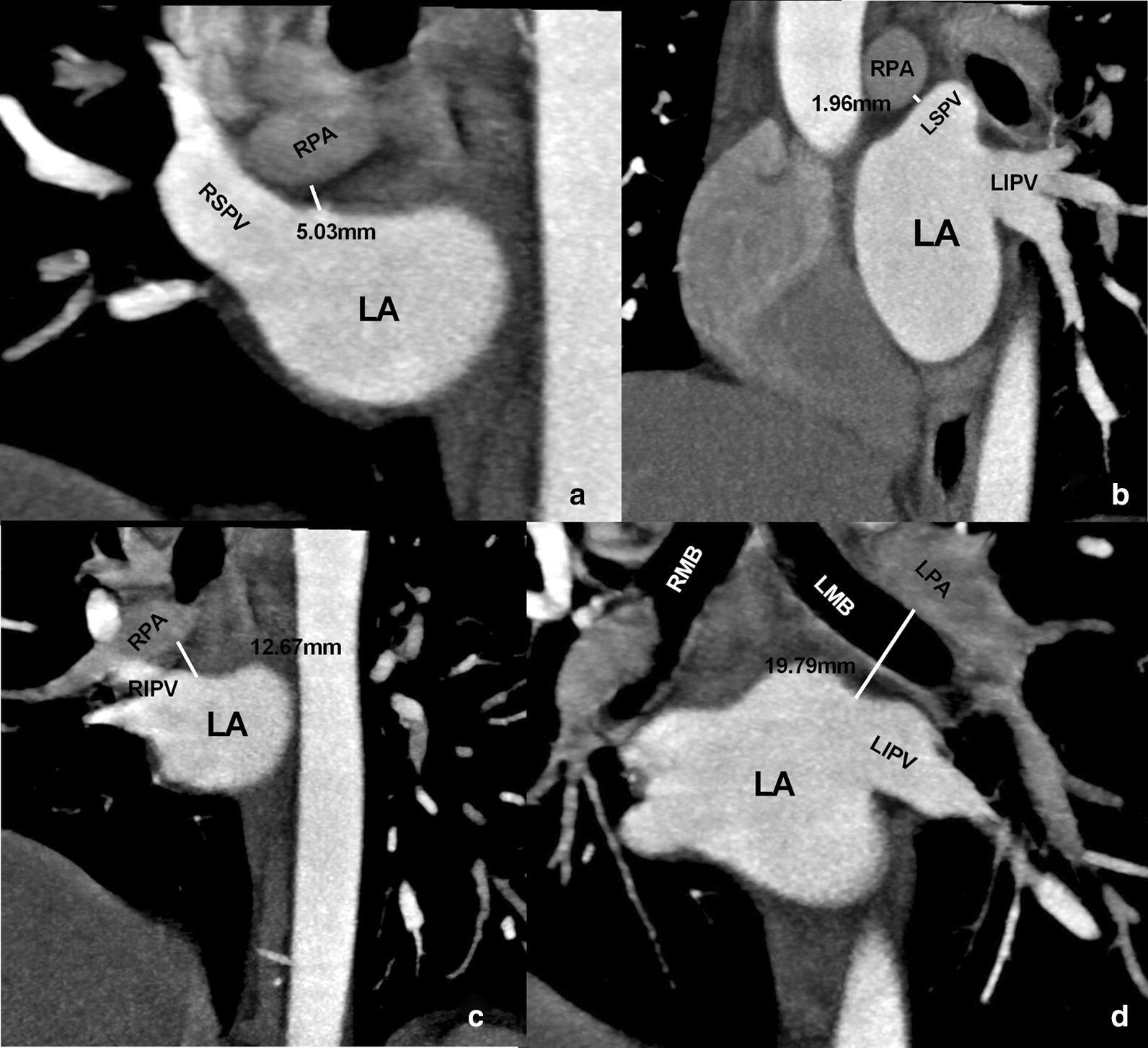
Measurement of the shortest spatial distance between the pulmonary vein (PV) orifices and the adjacent pulmonary arteries (PAs). **a**–**d** Shortest distance from the right superior, left superior, right inferior, and left inferior PV orifices to the adjacent PAs, respectively. LA, left atrium; LPA, left PA; RPA, right PA; RSPV, right superior PV; RIPV, right inferior PV; LSPV, left superior PV; LIPV, left inferior PV

The shortest spatial distances from the four PV orifices to the nearest PAs and bronchial trees were measured, respectively. Taking the measurement of the right superior PV orifice to the adjacent PA as an example, we firstly identified the right superior PV orifice on the transverse axial image, and the layer with the shortest distance to the PA was determined. Then, positioning the right superior PV orifice at this layer as a center, the cross-line technique was used to adjust the cross-lines from the axial, coronal, and sagittal views, respectively. The shortest distance between this point and its adjacent PA was determined. Secondly, we identified the right superior PV orifice on the sagittal and coronal images, respectively, and a similar cross-line technique was utilized to determine the shortest distance between this point and the adjacent PAs, respectively. Finally, the smallest value among the three measurements was selected as the final shortest spatial distance between the right superior PV orifices to the adjacent PAs. Based on the same measurement method described above, the shortest spatial distances were measured between the four PV orifices and the adjacent bronchi, as well as between the other three PV orifices and adjacent PAs (Fig. 4).

#### Measurement from the PV orifices to the contact points of the PV with PAs and bronchi

**Fig. 5:**
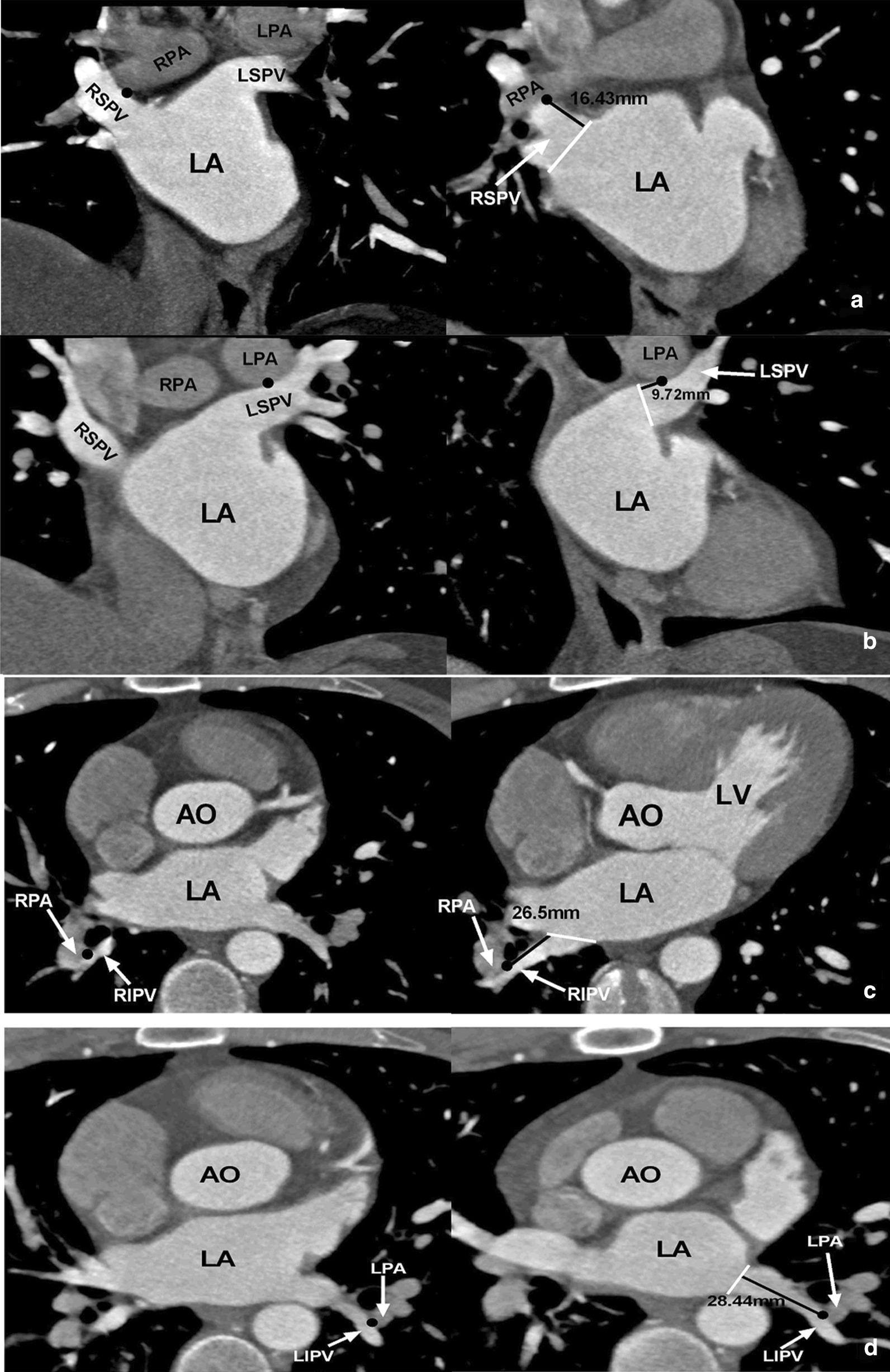
Measurement of the distances from the pulmonary vein (PV) orifices to the contact point between the PV and the pulmonary artery (PA). **a**–**d** Distances from the right superior, left superior, right inferior, and left inferior PV orifices to the contact points between the corresponding PV and PA, respectively. The left panel shows the images at the contact point level, and the right panel shows the distances between the contact points and the PV orifice. The black dot represents the contact point, the white line represents the cross-section of the PV orifice, and the black line represents the distances between the contact point and the PV orifice. AO, aorta; LV, left ventricle; LA, left atrium; LPA, left PA; RPA, right PA; RSPV, right superior PV; RIPV, right inferior PV; LSPV, left superior PV; LIPV, left inferior PV

Taking the measurement of the right superior PV and PA as an example, we firstly identified the contact point between the right superior PV and PA on the transverse axial image. Then, positioning the contact point as a center, cross-line technique was used to adjust the cross-lines from the axial, coronal, and sagittal views, respectively, and the shortest spatial distance from this point to the right superior PV orifice was recorded. Also, the shortest spatial distances from the right superior PV orifice to the contact points of the PV with the bronchi, as well as those from the other three PV orifices to the contact points of the PVs with PAs, and bronchi were measured (Fig. 5).

### Statistical methods

Data were analyzed using SPSS 22.0 statistical software (IBM Company, USA). Normal distribution data were expressed as mean ± standard deviation, and non-normal distribution data were expressed as median (interquartile range). The enumeration data (the ratio of groups in each interval) were expressed as percentage. Two groups of normal distribution samples were compared by independent sample (non-paired) *t* test. Rank sum test was used to compare two groups of samples with non-normal distribution. Chi-square test was used to compare the enumeration data. *α* = 0.05 with* P* < .05 was set as the statistically significant difference.

## Results

### Patient population

A total of 126 consecutive patients, including 67 men and 59 women, were enrolled in this study. The average age, height, and weight of the patients was 54.28 ± 9.28 years, 165.75 ± 3.23 cm, and 56.59 ± 10.92 kg, respectively.

### 3D reconstruction images of the LA, PAs, PVs, and bronchi

3D MSCT reconstructed images showed that four PVs originated from the dorsal side of the LA. The morphology and inner diameters of the PV orifices varied greatly among different individuals. The PA trunk and the left and right PAs were localized vertically above the LA roof. The courses of the right PAs across over the superior LA and were closely related to the LA roof. The left PAs were mainly located above the left superior PVs and behind the LA, and hence, were more distant to the LA roof than the right PAs. The bilateral inferior PVs were localized relatively far away from the left and right main PAs, but close to the ipsilateral PA branches, even directly contact with the distal segmental PAs in some patients. The left and right primary bronchi were localized vertically above the LA roof. The proximal part of the primary bronchi was far away from the LA roof and the PVs, while the distal part was close, even directly contact with the PVs in some patients (Fig. 1).

### LA size and diameter of the PV orifice

The mean LA transverse (LA1), vertical (LA2), and anteroposterior diameters (LA3) of 126 patients was 44.36 ± 5.56 mm (range 43.34–84.67 mm), 50.2 ± 5.56 mm (range 31.27–77.23 mm), and 44.15 ± 5.84 mm (range 24.22–53.76 mm), respectively. We used LA1 × LA2 × LA3 to denote the LA size, and the results showed that the LA size of 126 patients was 128,372 ± 40,466 mm^3^, which was significantly related to the height (*r* = 0.36, *P* < .01), and weight (*r* = 0.41, *P* < .01).

The mean diameters of the four PV orifices and the correlation of the diameter with the height and weight in patients were shown in Table 1.

**Table 1:** The mean diameters of the pulmonary vein orifices and the correlation of the diameter with height, weight

PV	Mean diameter (mm)	Correlation analysis	
		Height	Weight
RSPV	18.33 ± 2.77	*r* = 0.345,* P* < .001	*r* = 0.416, *P* < .001
RIPV	16.42 ± 2.50	*r* = 0.1,* P* = .297	*r* = 0.115, *P* = .225
LSPV	17.75 ± 2.88	*r* = 0.456, *P* < .001	*r* = 0.461,* P* < .001
LIPV	15.49 ± 1.92	*r* = 0.202, *P* = .035	*r* = 0.239,* P* = .001

*PV* pulmonary vein, *RSPV* right superior PV, *RIPV* right inferior PV, *LSPV* left superior PV, *LIPV* left inferior PV

### Distances from the LA roof to the adjacent bronchi and PAs

**Fig. 6:**
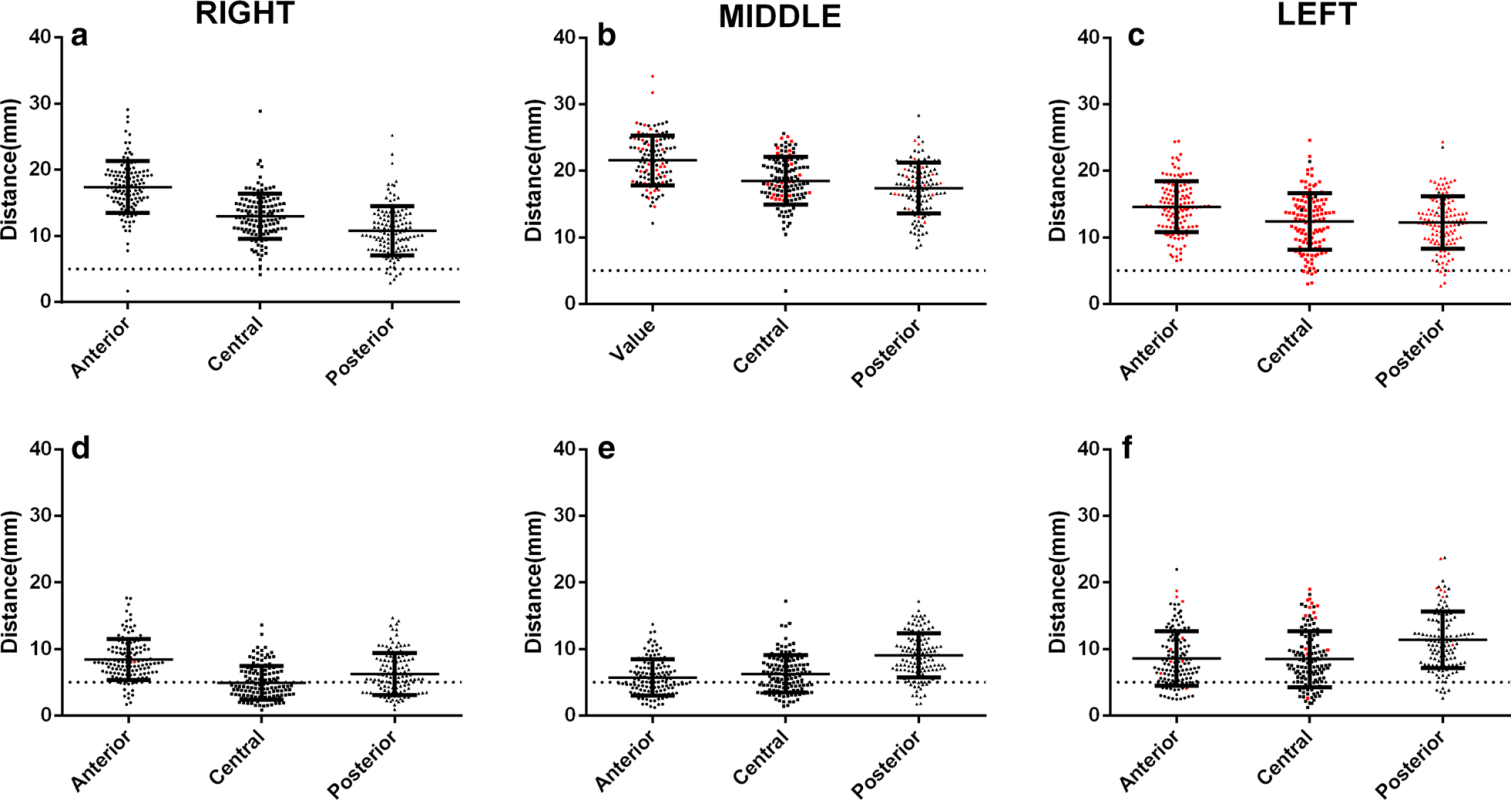
Distances from the nine points on the left atrial (LA) roof to the adjacent bronchus and pulmonary artery (PA). a–**c** Shortest spatial distances from the right, middle, and left points on the anterior, middle, and posterior layers of the LA roof to the adjacent bronchi, respectively. **d**–**f** Shortest spatial distances from the right, middle, and left points on the anterior, middle, and posterior layers of the LA roof to the adjacent PA, respectively. The black dots represent the distance closer to the right bronchus or PA, and the red dots represent the distance closer to the left bronchus or PA. The dotted line denotes the distance of 5 mm

The mean shortest spatial distances from the nine points of the LA roof to the adjacent primary bronchi were shown in Table 2 (Fig. 6).

**Table 2:** The mean shortest spatial distances and percentage of the distance < 5 mm from the points of the left atrial roof to the adjacent primary bronchi

Layer	Right (a)		Middle (b)		Left (c)	
	Distance (mm)	Distance < 5 mm (%, n)	Distance (mm)	Distance < 5 mm (%, n)	Distance (mm)	Distance < 5 mm (%, n)
Anterior	17.42 ± 3.93	0.79% (1/126)	21.60 ± 3.70	0% (0/126)	14.63 ± 3.82	0% (0/126)
Middle	12.98 ± 3.44	0.79% (1/126)	18.50 ± 3.59	0.79% (1/126)	12.38 ± 4.27	4.76% (6/126)
Posterior	10.80 ± 3.71	4.76% (6/126)	17.39 ± 3.81	0% (0/126)	12.25 ± 3.96	3.17% (4/126)
*P* value	.12	.04	.23	.37	.01	.06

The averaged minimum spatial distances from all the right points (1a, 2a, 3a), the midpoint (1b, 2b, 3b) and left point (1c, 2c, 3c) of the LA roof to the bronchi were 10.43 ± 3.41 mm, 16.95 ± 3.84 mm, and 2.71 ± 3.84 mm, respectively. No significant difference was observed in the shortest distances between the left and right points to the bronchi (*P* = .22), while significant differences were found between the midpoints and left points (*P* < .0001) and between the midpoints and right points to the bronchi (*P* < .001).

All the right points (100% points “a”) on the LA roof were closer to the right primary bronchi. All the left points (100% point “c”) on the LA roof were closer to the left primary bronchi. 22.5% (85/378) of the middle points (points “b”) were closer to the left primary bronchi, and 77.5% (293/378) of the middle points were closer to the right primary bronchi. The percentages of shortest spatial distance < 5 mm from nine points of the LA roof to the bronchi were shown in Table 2.

The mean shortest spatial distances from the nine points of the LA roof to the adjacent PA branches were shown in Table 3 (Fig. 6).

**Table 3:** The mean shortest spatial distances and percentage of the distance < 5 mm from the points of the left atrial roof to the adjacent pulmonary arteries

Layer	Right (a)		Middle (b)		Left (c)	
	Distance (mm)	Distance < 5 mm (%, n)	Distance (mm)	Distance < 5 mm (%, n)	Distance (mm)	Distance < 5 mm (%, n)
Anterior	8.46 ± 3.09	10.31% (13/126)	5.71 ± 2.73	46.83% (59/126)	8.57 ± 4.11	19.84% (25/126)
Middle	4.95 ± 2.52	58.73% (74/126)	6.29 ± 2.81	38.10% (48/126)	8.48 ± 4.22	20.63% (26/126)
Posterior	6.25 ± 3.16	39.68% (50/126)	9.09 ± 3.30	11.11% (14/126)	7.79 ± 3.92	4.76% (6/126)
*P* value	< .001	< .001	< .001	< . 001	< . 001	< .001

The averaged minimum spatial distances from all the right points (1a, 2a, 3a), the middle point (1b, 2b, 3b) and left point (1c, 2c, 3c) of the LA roof to the adjacent PAs were 4.65 ± 2.43 mm, 5.29 ± 2.56 mm, and 7.79 ± 3.92 mm, respectively. Significant differences were detected in the shortest distances between the left and right points (*P* = .041), the middle and left points (*P* < .001), and the middle and right points (*P* < .001) to the PAs.

All the right points (100% points “a”) and middle points (100% points “b”) on the LA roof were closer to the right PAs. A majority of the left points (91.8% points “c”) were closer to the right PAs, and only 8.20% (31/378) of the left points were closer to the left PAs. The percentages of the shortest spatial distance < 5 mm from the nine points of the LA roof to the PAs were shown in Table 3.

The correlation analysis showed that the shortest spatial distances from the right points on the anterior layer (1a) (*r* = 0.30, *P* < .05) and the middle layer (2a) (*r* = 0.25, *P* < .05) of the LA roof to the bronchi were correlated with the LA size. The shortest spatial distance from the right points on the anterior layer (1a) (*r* = 0.23, *P* < .05), the left points on the anterior layer (1c) (*r* = 0.19, *P* < .05), the left points on the middle layer (2c) (*r* = 0.21, *P* < .05), and the right points on the posterior layer (3a) (*r* = 0.21, *P* < .05) to the PAs were correlated with the LA size.

### Shortest spatial distances between the pulmonary vein orifices and bronchi, PAs

**Fig. 7:**
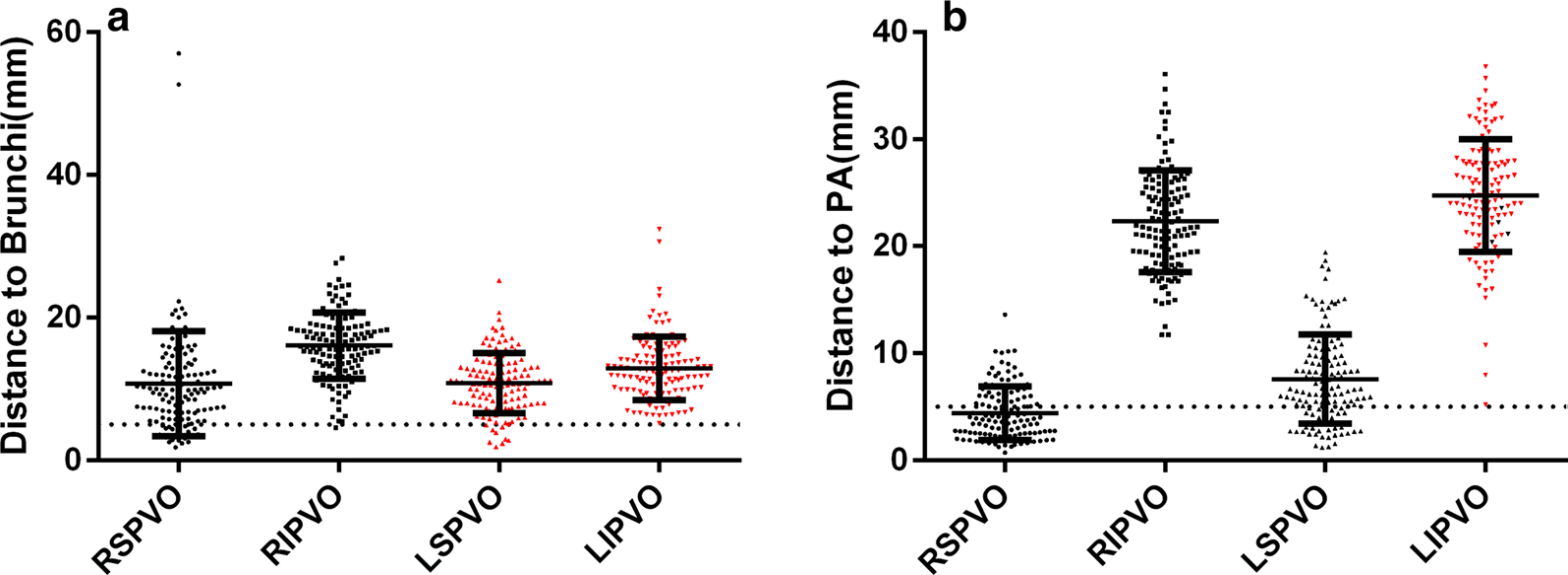
Distances from the pulmonary vein (PV) orifice to the bronchi and pulmonary arteries (PAs). **a** Shortest spatial distances between the four PV orifices and the bronchial tree. **b** Shortest spatial distance between the four PV orifices and the PAs. The black dots indicate the distance from the PV orifice to the right bronchus or right PA, and the red dots indicate the distance from the PV orifice to the left bronchus or left PA. The dotted line denotes the distance of 5 mm

The minimum distances and percentage of the distance < 5 mm from the four PV orifices to the adjacent bronchi and PAs were shown in Table 4 (Fig. 7).

**Table 4:** The minimum spatial distances and percentage of the distance < 5 mm from the four pulmonary vein orifices to the bronchi and pulmonary arteries

Pulmonary vein orifices	Bronchi		Pulmonary arteries	
	Distance (mm)	Distance < 5 mm (%, n)	Distance (mm)	Distance < 5 mm (%, n)
Left superior	10.82 ± 4.19	7.14% (9/126)	7.59 ± 4.14	26.98% (34/126)
Left inferior	12.87 ± 4.46	0% (0/126)	24.74 ± 5.26	0% (0/126),
Right superior	10.73 ± 7.34	15.9% (20/126)	4.43 ± 2.51	63.49% (80/126)
Right inferior	16.06 ± 4.63	0.79% (1/126)	22.33 ± 4.75	0% (0/126)
*P* value	< .001	< .001	< .001	< .001

The two superior PV orifices were closer to the PAs than the two inferior PV orifices (*P* < .001). The data of the left and right superior PVs were therefore combined together to compare with the combined left and right inferior PVs. The results showed that the averaged shortest distances from the two superior and inferior PV orifices to the adjacent PAs were 6.01 ± 3.77 mm and 23.53 ± 5.14 mm, respectively (*P* < .001). In comparison to the left superior PV orifices, the right superior PV orifices were more closely related to the PAs (*P* < .001). Moreover, 100% patients were presented with the left superior, right superior and inferior PV orifices closer to the right PAs than the left PAs, and all the patients had the left inferior PV orifices closer to the left PAs than the right PAs (Fig. 7).

The correlation analysis showed that the shortest spatial distance from the right superior PV orifices to the PAs was related to the LA size (*r* = − 0.219, *P* = .014). There was a correlation between the diameter of the left superior PV orifices and the distance of the PV orifices to the bronchi (*r* = 0.274, *P* = .002).

### Distances from PV orifices to the contact points between the PVs and PAs, bronchi

**Fig. 8:**
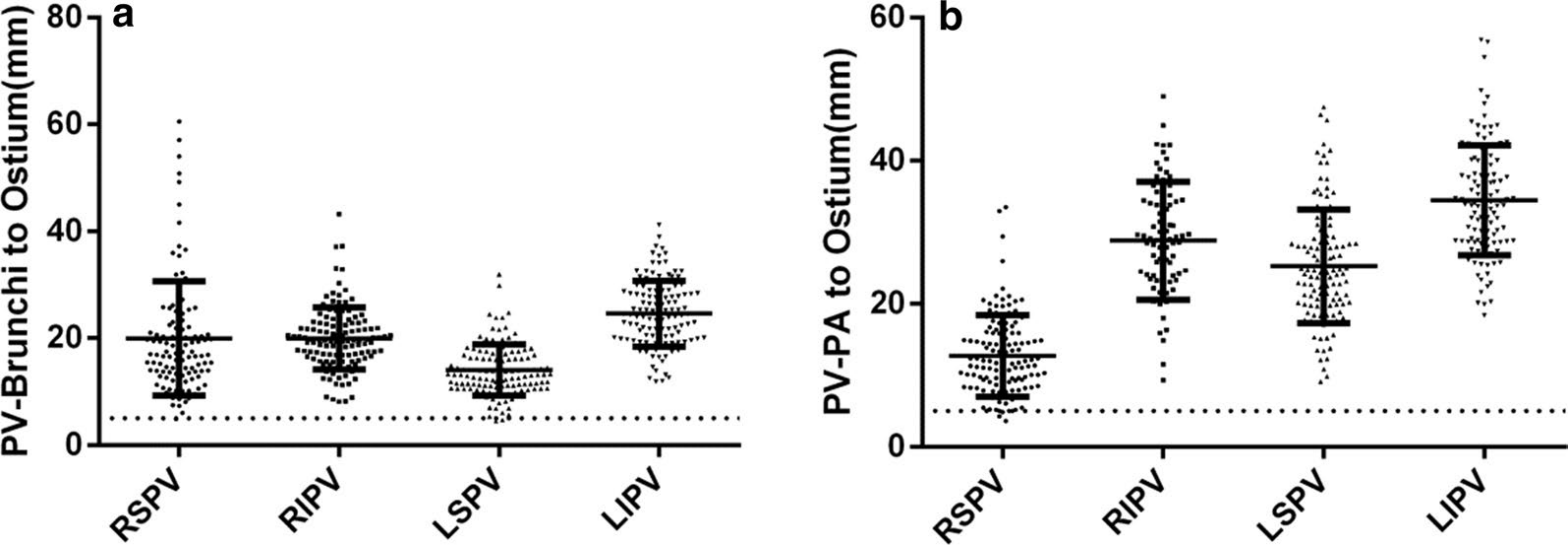
Distances from the pulmonary vein (PV) orifices to the contact points between the PV and the adjacent bronchus or PA. **a** Distances from the PV orifices to the contact points between the PV and bronchus. **b** Distances from the PV orifices to the contact points between the PV and PA

Fourteen of 126 patients (11.1%) did not have a direct contact between the right superior PVs and the adjacent bronchi and branches within the visible windows of view. The distances from the left superior and inferior, right superior and inferior PV orifices to the corresponding contact points between the PVs and bronchi were 14.07 ± 4.79 mm, 24.61 ± 6.18 mm, 19.99 ± 10.68 mm, and 19.96 ± 5.79 mm (*P* < 0.05), respectively. The ratio of the distances < 5 mm from the left superior and inferior, right superior and inferior PV orifices to the corresponding contact points between the PVs and bronchi were 1.59% (2/126), 0% (0/126), 0.89% (1/112), and 0% (0/126), respectively (Fig. 8).

PA branches had no direct contact with the left superior and inferior, right superior and inferior PVs in 5.6% (7/126), 34.1% (43/126), 0% (0/126), and 7.9% (10/126) of the patients, respectively. The distances from the left superior and inferior, right superior and inferior PV orifices to the corresponding contact points of the PVs and PAs were 25.27 ± 7.95 mm, 34.49 ± 7.71 mm, 12.7 4 ± 5.71 mm, and 28.81 ± 8.25 mm (*P* < .001), respectively. The ratio of the distances < 5 mm from the left superior and inferior, right superior and inferior PV orifices to the corresponding contact points between the PVs and PAs were 0% (0/119), 1.20% (1/83), 3.97% (5/126), and 0% (0/116) (*P* = 0.02), respectively (Fig. 8).

There is a significant correlation between the diameters of right inferior PV and the distances from the PV orifices to the contact points of the PVs and bronchi (*r* = 0.047, *P* = .599). The diameters of the right superior PVs (*r* = 0.215, *P* = .015) and left superior PVs (*r* = 0.239, *P* = .09) were significantly correlated to the distances from the PV orifices to the corresponding contact points of the PVs and PAs.

## Discussion

In recent years, with the rapid development of radiofrequency catheter and balloon cryoablation techniques for AF, the damage caused by the ablation energy to the tissues surrounding the ablation targets has attracted attention and has been investigated in-depth with respect to cardiac morphology and anatomy. Previous studies showed that tissue structures < 5 mm away from ablation targets had a high risk of damage [15]. Anatomically, the AF ablation targets, such as PV orifice and LA roof, are closely adjacent to the trachea/bronchial tree and PAs and their branches. Therefore, the ablation energy might damage the adjacent bronchi and PAs, resulting in cough, dyspnea, hemoptysis, and other symptoms [5, 9]. Verma et al. [14] observed in 7 patients with the intraoperative real-time bronchoscopy that ice crystals and internal hemorrhage were formed within the left primary bronchi during the cryoablation of the left superior PVs. Kuck et al. [16] reported that the incidence of chest pain, cough, and hemoptysis in AF cryoablation and radiofrequency ablation was 2.9% and 1.9%, 0.8% and 0.5%, 0.3% and 0.5%, respectively.

Recently, the temporal and spatial resolution of MSCT has been greatly improved, which in turn, displayed the structures of the heart, mediastinum, and blood vessels more clearly and accurately. The unique 3D reconstruction technique of MSCT displayed the anatomy of the heart and adjacent tissues from variable angles and directions, thereby providing a powerful tool for exploring the anatomy correlation of the heart for arrhythmia ablation. The unique MPR post-processing technique of MSCT allows for an accurate measurement of the shortest spatial distance between two adjacent tissues in vivo. In the present study, we used the MPR post-processing technique of MSCT to quantitatively evaluate the anatomical adjacency of the AF ablation targets, such as four PV orifices and the LA roof, to the surrounding airway and the PA system, in order to provide guidance for cardiac electrophysiologists before ablation to reduce the complications of AF intra-operatively and post-operatively.

### Anatomical correlation between the LA roof and bronchi, PA

In this study, we artificially divided the LA roof into three layers from forward to backward, and each layer was divided into three points from right to left. The results showed that the shortest spatial distances from the nine measuring points to the adjacent bronchi and PAs were different on the anterior, middle, and posterior layers of the LA roof. The differences were not only reflected in the average distances at different layers but also in the subset of patients who were categorized as beyond the “safe distance.” Among the nine measuring points of the LA roof, the anterior points (right, middle, and left) presented longer distances and smaller proportion of the distance < 5 mm to the adjacent bronchi than the middle or posterior points. Therefore, it might be reasonable to adopt the ablation targets at the anterior position of the LA roof, in order to reduce the bronchial mucosa injury. In this study, we found that there were a few patients had the shortest distance < 5 mm from the LA roof to the bronchi, which was different from Li et al. [17] reporting that 100% patients had a long distance from the LA roof to the primary bronchi, and hence, the ablation of the LA roof was unlikely to damage the bronchi. The main limitation of the study by Li et al. is that they measured the distances at only one layer of the LA roof to the bronchi. Since the LA roof was not a straight line, but a 3D curved dome surface, it is better to measure the points of the LA roof at the anterior, middle, and posterior levels. Since the middle and posterior layers of the LA roof were more closely related to the bronchi than the anterior layers, and the distances from the middle posterior points to the adjacent bronchi were < 5 mm in some patients, the authors presumed that ablation at the LA roof, especially the posterior location, might cause airway damage in some patients. Therefore, it might be reasonable to adopt the ablation targets at the anterior position of the LA roof as far as possible, in order to reduce the bronchial mucosa injury.

The present study found that the distances from the LA roof to the PAs were shorter than those to the bronchi, indicating that PAs had a closer anatomical relation with the LA roof than the bronchi. Since a large subset of patients had the shortest spatial distance < 5 mm from the points of the LA roof to the PAs, it is presumed that irrespective of the ablation at the anterior, middle, or posterior positions of the LA roof, it might have possibilities damaging the adjacent PA branches. However, clinical cases of ablation-related PAs damage were not reported currently in literatures, which may be ascribed to the ample bloodstream in PAs that might greatly dissipate the energy of ablation.

The present study showed that 100% of the right points and 77.5% of the midpoints of the LA roof were closer to the right bronchi, while 100% of the left points and 22.5% of the midpoints of the LA roof were closer to the left bronchi. Interestingly, we found that only 8.2% of the left points of the LA roof were closer to the left PAs, while 91.8% of the left points of the LA roof were closer to the right PAs. Based on the reconstructed 3D MSCT images, we knew that the courses of the right PAs across almost of the entire LA roof, while the left PAs were mainly located above the left superior PVs and behind the LA; thus, the anatomical correlation between the right PAs and the LA roof was closer than that of the left PAs.

### Anatomical correlation between PV orifices and bronchi, PAs

The PVs had a close anatomical relation to the PAs and bronchi, which are accompanied by PAs and bronchi at the hilum of lung. Because the PV vestibules are the most common targets for AF ablation, we quantitatively measured the minimum spatial distance between the four PV orifices and the adjacent PAs and bronchi. The results of this study were consistent with those by Wu et al. and Li et al. [17, 18] showing that the superior PV orifices are closer to the PAs and bronchi than the inferior PV orifices. In addition, we found that 7.14% of the left superior PV orifices and 15.9% of the right superior PV orifices were located with a distance < 5 mm to the bronchi. Verma et al. [14] reported the phenomenon of freezing ice formation in the bronchus during the ablation of the left superior PV, while Velghe et al. [19] reported the cases of atrial-right bronchial fistula after right superior PV ablation. The present study revealed that both the left and right superior PVs had the distances < 5 mm to the bronchi in a proportion of patients, and hence, the ablation of either left or right superior PV might have possibilities of damaging the adjacent bronchi. Although the incidence of atrial-bronchial fistula was low and only few cases were reported in the literature, the high mortality warrants additional attention for the cardiac electrophysiologists.

Consistent with the results of Li et al. [17] we found that the anatomical correlation of bilateral superior PV orifices to PAs was closer than that of bilateral inferior PV orifices. In comparison with the left superior PV orifices, the right superior PV orifices were more closely related to the PAs, evidenced by 27% of the left and 63% of the right superior PV orifices had the distances of < 5 mm to the adjacent PAs. Based on the close anatomic adjacency between PV orifices and PAs, the superior PVs, especially the right superior PV ablation might have possibilities of risking the adjacent PA branches. However, currently there were no pathological or autopsy reports confirming the damage of the PAs during AF ablation, which may be associated with the ample bloodstream in the PAs that dissipate the ablation energy.

### Distances from PV orifices to the contact points between the PV and PAs, bronchus

The PVs had a direct contact with the bronchial tree and PA branches in the majority of the patients. We found that the nearest contact point between PVs and PAs or bronchial branches were distant from each of the corresponding PV orifices. The result of only few patients had the distance < 5 mm from the upper PV orifice to the contact points in the present study was not in agreement with the reports by Wu et al. [18] that the contact points were close to the PV orifices in most patients (< 5 mm).

### Clinical significance

During the radiofrequency or balloon cryoablation of AF, it is necessary to pay enough attention to protecting the important adjacent organs around the ablation targets. This study explored the anatomical relation of the common AF ablation targets of the four PV orifices and the LA roof with the adjacent airway and PAs. The findings in the present study suggested that it might have a higher possibility of damaging the bronchial tree if the mid-posterior LA roof was targeted during AF ablation. The ablation of either left or right superior PVs might have possibilities of damaging the adjacent bronchi. In anatomy evidenced by MSCT, the PAs had a closer anatomical relationship with the LA roof and PV orifices than the bronchi. However, clinical cases of ablation-related PAs damage were not currently reported, which might ascribe to the ample bloodstream in PAs that dissipate the ablation energy. The authors proposed that the variable anatomical proximity among individuals makes it difficult to establish the same protective strategies for every patient of AF ablation Thus, doctors should take full advantage of the robust 3D reconstruction of MSCT and the powerful MPR technique to conduct a comprehensive preoperative anatomical assessment of the ablation targets, in order to establish different individualized ablation strategies for every patient to reduce the complications of AF ablation. If combined with high‐power short‐duration radiofrequency ablation, which lesion diameters were significantly larger and lesion depths were significantly smaller than standard settings [20, 21], the individualized ablation strategies maybe further reduce the incidence of complications.

To date, MSCT angiography has been routinely applied in the patients before AF ablation. The MSCT images of the bronchial tree and PAs in the present study were acquired along with the clinical routine coronary CTA imaging, atrial and PV imaging, esophageal and phrenic nerve imaging et al. which neither requires an increased X-ray radiation nor the dose of the idolized contrast agent. This “one-stop” CT examination enabled to assess the anatomical correlation of the ablation targets, which provides guidance for cardiac interventionist to reduce the complications of AF ablation.

### Limitations

Firstly, the patients enrolled in this study were inpatients in our hospital with routine coronary MSCT angiography. Since the MSCT angiography is not an optimal option for evaluating the coronary artery stenosis under irregular heart rhythm, patients with AF undergoing radiofrequency ablation or balloon cryoablation were not enrolled in this study. Whether or not the anatomical adjacency of the ablation targets of the PVs and LA roof with bronchi and PAs in AF patients was same as that was described in this study remains to be investigated. Secondly, respiratory and cardiac movements might affect the morphology of the bronchi and atrium, thereby reducing the accuracy of evaluating the anatomical proximity among the LA, PVs, adjacent airways, and PAs. The measurement of the spatial distances under different respiratory motion status and different RR intervals might better clarify the precise anatomical correlation between the ablation targets and the adjacent bronchi and PAs. Thirdly, the sample size of this study was relatively small, and the influence of age, gender, body mass index, heart disease history, and heart rate on the distances between the ablation targets and adjacent bronchi and PAs was not elaborated. We planned to utilize the noninvasive method of MSCT imaging with larger sample size in the future to confirm our conclusions, to further evaluate the relevant risks of AF ablation, as well as to explore the protective therapeutic strategies for AF ablation.

## Conclusions

MSCT angiography is a feasible method to assess the anatomical correlation between the ablation targets of AF and adjacent airway and PA systems. The PAs had a closer anatomical relationship with the LA roof and PV orifices than the bronchi. Compared with the inferior PVs, the superior PVs, especially the right superior PVs, were more closely related to the bronchi and PAs. The ablation of the middle and posterior position of the LA roof might have higher possibilities of risking the adjacent bronchi. Coronary MSCT angiography is a valuable tool to evaluate the anatomical adjacency of the ablation targets, which provides reference for cardiac electro-physiologists to reduce the AF ablation complications.

## Data Availability

The datasets used and/or analyses during the current study are available from the corresponding author on reasonable request.

## Abbreviations

AF: Atrial fibrillation
PV: Pulmonary vein
LA: Left atrium
PA: Pulmonary artery
Pas: Pulmonary arteries
STOP-AF: Sustained treatment of paroxysmal atrial fibrillation
MSCT: Multi-slice spiral computed tomography
VR: Volume rendering
MPR: Multi-planar reformation
3D: Three-dimensional
